# The prognostic value of lymph nodes count on survival of patients with node-negative gastric cancer

**DOI:** 10.18632/oncotarget.9845

**Published:** 2016-06-06

**Authors:** Wei-feng Zheng, Ting-ting Ji, Yong Lin, Rong-zhou Li

**Affiliations:** ^1^ Department of Gastroenterology, Rui'an People's Hospital, Third Affiliated Hospital of Wenzhou Medical University, Wenzhou 325200, Zhejiang Province, China

**Keywords:** gastric cancer, lymph node count, survival analysis, SEER

## Abstract

The retrieved lymph node (LN) count has been validated as a prognostic factor in various cancers. However, the interaction between LN counts and patients' prognosis in gastric cancer with negative-LN metastasis is not fully studied. Surveillance, Epidemiology, and End Results Program (SEER)-registered gastric cancer patients were used for analysis in this study. Patients operated on for gastric cancer at N0 stage between 2004 and 2012 were identified from the SEER database. The association between the LN counts and survival was assessed using the Cox proportional hazards model. Patients were stratified into 1–4, 5−13, and > 13 subgroups according to LN count cut-off values determined by X-tile program, with the 5-year cause specific survival (CSS) rate of 64.8%, 72.5%, and 79.4%, respectively. LN count was also validated as an independently prognostic factor in multivariate Cox analysis (*P* < 0.001). In addition, nomograms including LN counts on CSS were established according to all significant factors, and the c-index was 0.703 (95% CI: 0.672−0.734). Further study indicated that patients with no LN metastasis had a decreased risk of death for each patient with LN examined up to approximately 14 LNs. Collectively, our study firmly demonstrated that the number of the retrieved LNs count was an independent prognostic factor for gastric cancer with no LN metastasis. The higher the LN count, the better the survival would be; the best CSS was observed on the LN count more than 14.

## INTRODUCTION

Although the incidence of gastric cancer has been substantially declining for several decades, it remains a major cause of cancer mortality because of its poor prognosis [1]. Complete resection remains the only treatment that can lead to cure for gastric cancer and the presence or absence of lymph node (LN) metastasis was widely considered to be the most important predictor for gastric cancer survival, and LN-negative patients have been shown to have better survival than those with LN metastasis [2, 3]. However, patients with node-negative gastric cancer also experience recurrences and may have fatal outcomes [2, 4, 5]. For such reasons, many researchers make efforts to investigate the prognostic factors related to recurrence and survival.

The tumor-node-metastasis (TNM) classification of the Union for International Cancer Control (UICC) for gastric cancer is considered as the best classification system because of its ability to provide precise prognostic estimation and guidance for patients through appropriate therapeutic programs. Currently, the 7th edition TNM classification is most widely used for the staging of gastric cancer, but it does not define the minimum number of retrieved LNs necessary, especially for the gastric cancer patients with no LNs metastasis. Some previously published articles sought to investigate the prognostic value of LNs retrieval in node-negative gastric cancer, but the number of patients included in their study was small [6, 7], and the cutoff used in the study may also be arbitrary.

The objective of this study was to investigate prognosis value of retrieved LNs count and other clinical pathologic characteristics in patients who had no LNs metastasis, and to find a reasonable retrieved LNs cutoff value that guarantee accurate TNM stage. In order to get convincing results in a larger series patients, we using SEER (Surveillance, Epidemiology and End Results)-registered database to analyze and X-tile program [8] to determine the optimal cutoff.

## RESULTS

### Patient characteristics

Of the 5,794 patients (62.2% male, 37.8% female) with LN negative gastric cancer who met the eligibility criteria, 4,670 (80.26%) were diagnosed as adenocarcinoma, 907 (15.65%) as signet ring cell carcinoma, and 217 (3.75%) as mucinous adenocarcinoma. Most patients (65.9%) were white, and nearly half patients (50.7%) had poor- or undifferentiated tumors. Patients' demographics and pathological features are summarized in Table 1.

**Table 1:** Demographic and tumor characteristics of patients with node negative gastric cancer

	LNs Subgroup						χ^2^ Value	*P* Value
	1–4		5–13		> 13			
	*n* = 923		*n* = 2,332		*n* = 2,539			
Characteristic	No.	%	No.	%	No.	%		
Sex							3.560	0.169
male	590	63.9%	1466	62.9%	1545	60.9%		
female	333	36.1%	866	37.1%	994	39.1%		
Age	72 (62–80)		70 (60–78)		66 (57–75)			< 0.001^**^
Race							31.777	< 0.001
Caucasian	624	67.6%	1600	68.6%	1596	62.9%		
Black	120	13.0%	262	11.2%	282	11.2%		
Other^*^	179	19.4%	470	20.2%	659	26.0%		
Married staus								
Married	541	58.6%	1444	61.9%	1653	65.1%		
Unmarried	347	37.6%	816	35.0%	791	31.2%		
Unknown	35	3.8%	72	3.1%	95	3.7%		
Pathological grading							12.562	0.014
High/Moderate	433	47.0%	1000	42.9%	1056	41.6%		
Poor/Anaplastic	421	45.6%	1190	51.0%	1327	52.3%		
Unknown	68	7.4%	142	6.1%	156	6.1%		
Histotype							2.093	0.351
Adenocarcinoma	748	80.7%	1899	81.4%	2026	79.8%		
Mucinous/Signet ring cell	178	19.3%	554	18.6%	513	20.2%		
Tumor size (mm)							103.516	< 0.001
< = 20	323	35.0%	765	32.8%	731	28.9%		
20–50	321	34.8%	891	38.2%	908	35.8%		
> 50	112	12.1%	371	15.9%	607	23.9%		
Unknown	167	18.1%	305	13.1%	290	13.1%		
Stage							26.923	< 0.001
I	608	65.9%	1499	64.3%	1521	59.9%		
II	274	29.7%	766	32.8%	943	37.1%		
III	38	4.1%	56	2.4%	67	2.6%		
Unknown^***^	3	0.3%	11	0.5%	8	0.3%		

* Other includes American Indian/Alaska native, Asian/Pacific Islander, and unknown.

** Mann-Whitney *U* test.

*** Unknown: undefined T stage.

**Figure 1:**
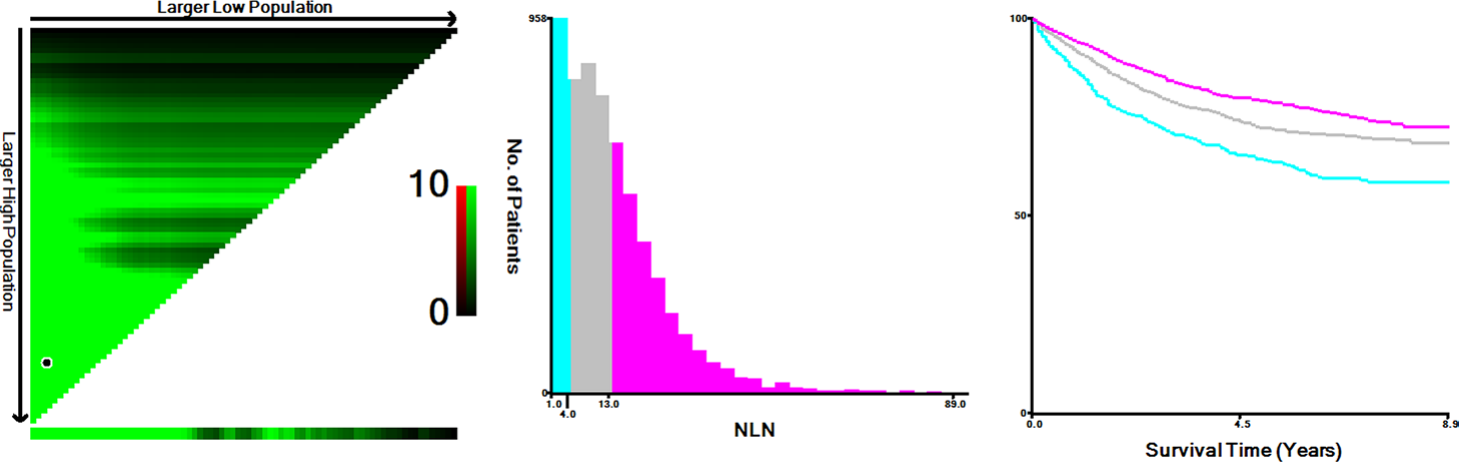
X-tile analysis of survival data from the SEER registry X-tile analysis was performed using data from SEER database. The sample of patients with gastric cancer was equally divided into training and validation sets. X-tile plots of the training sets are shown in the *left panels*, with plots of matched validation sets shown in the *smaller inset*. The optimal cut-point highlighted by the *black circle* in the *left panels* is shown on a histogram of the entire cohort (*middle panels*), and a Kaplan-Meier plot (*right panels*). *P* values were determined using the cutoff point defined in the training set and applying it to the validation set. *Figure 1* shows the optimal cutoff points for the lymph node negative patients (number of 4 and 13, χ^2^ = 72.026, *P* < 0.001).

All patients had at least one LN examined. The median number of LNs examined was 12 (IQR 7–19). The 5-year cause specific survival (CSS) was 74.0%. X-tile plots were constructed and the maximum of χ^2^ log-rank value of 72.026 was achieved when applying 4 and 13 as the cutoff values of retrieved LN number. Patients can be accordingly divided into the high, middle and the low subsets in terms of 5-year CSS, which were 64.8%, 72.5% and 79.4%, respectively (*P* < 0.001, Figure 1).

### Impact of total number of LNs examined on risk of death

Using Kaplan-Meier estimates, beside of the number of retrieved LNs, some other clinicopathological factors, including age (*P* < 0.001), race (*P* < 0.001), pathological grade (*P* < 0.001), tumor size (*P* < 0.001), and T stage (*P* < 0.001) were also found to be risk factors for CSS.

Further multivariate analysis showed that all six factors were associated with survival (Table 2). Elder patients (HR 0.90, 95% CI 0.87 to 0.93) had 1.593–fold increase in the risk of death. Non-Caucasian/Black patients (referent, Caucasian patients: HR 0.740, 95% CI 0.631–0.869) had an improved overall survival. Patients with poor/anaplastic tumor grade had a worse prognosis compared with tumors with high/moderate differentiation (HR: 1.380, 95%CI 1.219 to 1.564, high/moderate differentiation as reference). Patients with a T2 tumor had a 1.495-fold increase in the risk of death (HR 1.495; 95% CI 1.223 to 1.828); patients with T3 tumors had 2.770-fold increased risk (HR 2.770; 95% CI 2.341–3.277); T4a tumor had 3.731-fold increased risk (HR 3.731; 95% CI 3.058–4.553); and T4b tumor had 5.619-fold increased risk (HR5.619; 95% CI 4.324–7.302, T1 stage as reference). Tumor size was associated with survival as well (20 to 50 mm, HR 1.240; 95% CI 1.042–1.476; >50 mm, HR 1.402; 95% CI 1.154–1.703; <20 mm as reference). As anticipated, increasing LNs retrieval was associated with improved survival (5–13, HR 0.684, 95% CI 0.589–0.794; > 13, HR 0.501, 95% CI 0.428–0.587; 1–4 as referenced).

**Table 2:** Univariate and multivariate survival analyses for evaluating the influence of the number of LNs retrieved on CSS in node negative gastric cancer

Variable	5-year RCCS	Univariate analysis		Multivariate analysis	
		Log rank *χ^2^* test	*P*	HR (95% CI)	*P*
Sex		1.864	0.172		NI
Male	73.1%				
Female	75.8%				
Age		37.652	< 0.001		< 0.001
≤ 60	79.1%			Reference	
> 60	72.0%			1.593 (1.383–1.834)	
Race		34.396	< 0.001		0.001
Caucasian	71.3%			Reference	
Black	75.8%			0.918 (0.763–1.104)	0.362
Others	81.3%			0.740 (0.631–0.869)	< 0.001
Grade		57.923	< 0.001		< 0.001
High/Moderate	78.8%			Reference	
Poor/Anaplastic	69.3%			1.380 (1.219–1.564)	< 0.001
Unknown	81.0%			0.995 (0.745–1.330)	0.974
Histotype		3.390	0.066		NI
Adenocarcinoma	74.8%				
Mucinous/signet ring cell	71.4%				
Tumor size (mm)		149.109	< 0.001		0.007
< 20	84.4%			Reference	
20–50	70.9%			1.240 (1.042–1.476)	0.015
> 50	62.9%			1.402 (1.154–1.703)	0.001
Unknown	74.4%			1.295 (1.048–1.599)	0.016
T Stage		511.603	< 0.001		< 0.001
T1	86.3%			Reference	
T2	78.5%			1.495 (1.223–1.828)	< 0.001
T3	61.4%			2.770 (2.341–3.277)	< 0.001
T4a	52.2%			3.731 (3.058–4.553)	< 0.001
T4b	41.2%			5.619 (4.324–7.302)	< 0.001
Tx	26.1%			4.941 (2.812–8.680)	< 0.001
No. of LNs		72.026	< 0.001		< 0.001
1–4	64.8%			Reference	
5–13	72.5%			0.684 (0.589–0.794)	< 0.001
> 13	79.4%			0.501 (0.428–0.587)	< 0.001

NI: not included in the multivariate survival analysis.

**Figure 2:**
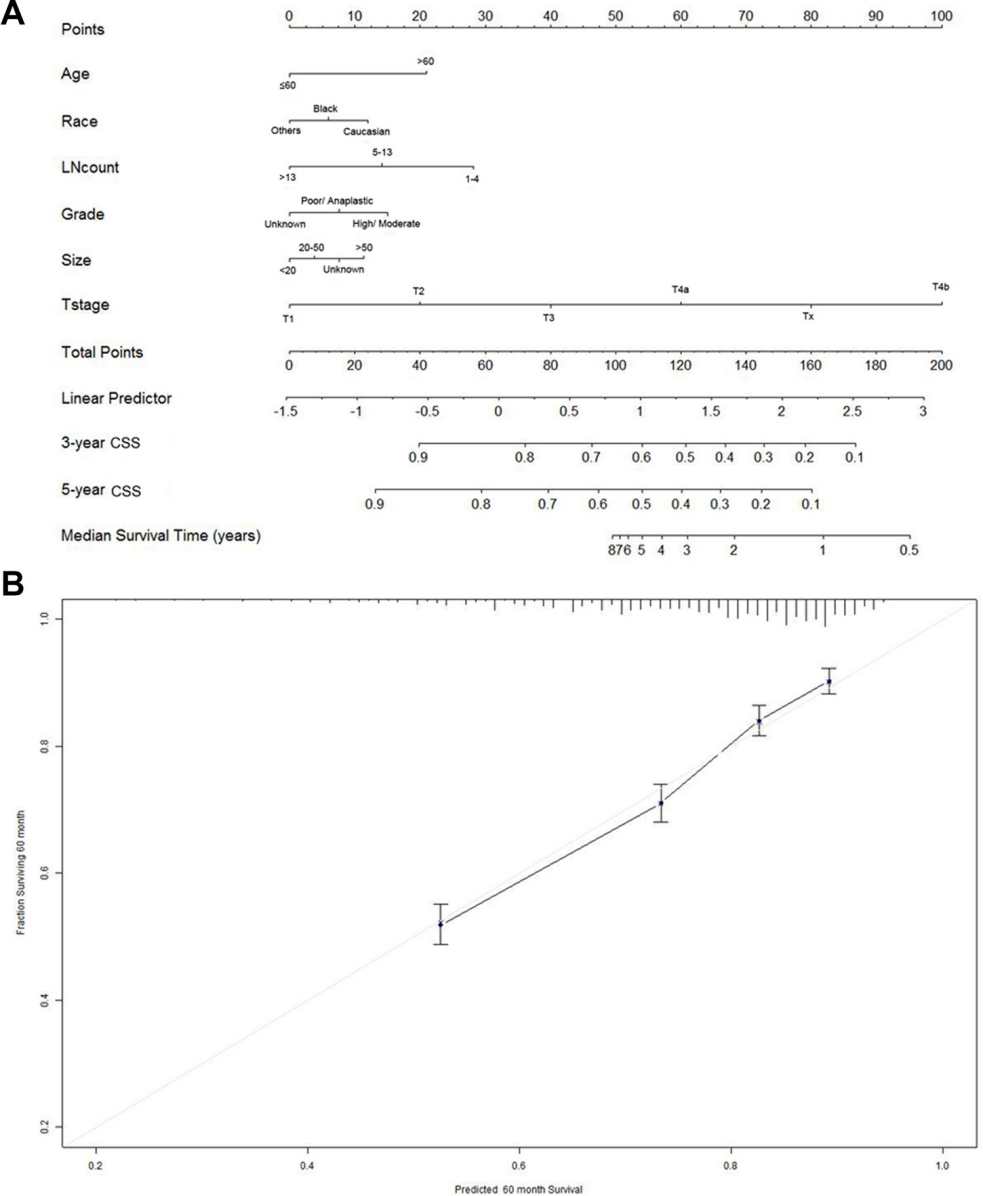
Nomogram for predicting 3- and 5-year cause specific survival (CSS) of gastric cancer patients (**A**) Using nomograms with clinicopathological characteristics are shown. Instructions for use of the nomogram: First, assign the points of each characteristic of the patient by drawing a vertical line from that variable to the points scale. Then, sum all the points and draw a vertical line from the total points scale to the 3- and 5-year CSS to obtain the probability of death. (**B**) The reference line is 45 degree and indicates perfect calibration.

The Cox model performance was composed of two components, discrimination and calibration. The ability of a model to separate subject outcomes is known as discrimination. Discrimination was quantified with the Harrell's concordance index (C-index), which is similar with the area under the receiver operating characteristic (ROC) curve but appropriate for censored data. As shown in Table 2, the C-index of Cox model is 0.703 (95% CI: 0.672–0.734). Calibration was performed by comparing predicted probability of CSS versus actual CSS on the 5,794 patients. The calibration curves for the probabilities of 3- and 5-year CSS showed excellent agreement of the nomogram prediction with actual observation as demonstrated in Figure 2.

### Identification of minimum number of retrieved LNs

To assess the influence of different cutoff points on gastric cancer CSS, we further analyzed the number of retrieved LN counts from 2 to 22. The 5-year CSS of patients with *n* (cutoff point) or more nodes and less than *n* nodes were calculated, respectively. The survival rate of patients with *n* or more nodes increased gradually when *n* ranged from 2 to 14. Patients with 14 or more LNs evaluated had a relative reduction of 16.9% for death from gastric cancer compared to those with two less LNs evaluated (79.4% versus 62.5%). After the number of 14, the survival rates were roughly equal (Table 3). It is likely that 14 is the minimum number of LNs that should be retrieved, above which the influence of negative LNs (NLNs) count on survival is minimal.

**Table 3:** Univariate analysis for the influence of different cutoffs on CSS in node-negative gastric cancer

Cutoff	No.	5-year CCS	Log-rank χ^2^	*P* value
< 2	204	62.5%	16.291	< 0.001
≥ 2	5590	74.5%		
< 3	544	63.7%	31.843	< 0.001
≥ 3	5339	75.0%		
< 4	674	63.9%	49.099	< 0.001
≥ 4	5120	75.5%		
< 5	923	64.8%	55.491	< 0.001
≥ 5	4871	75.9%		
< 6	1165	66.3%	50.457	< 0.001
≥ 6	4629	76.2%		
< 7	1447	65.9%	64.626	< 0.001
≥ 7	4347	77.1%		
< 8	1704	66.6%	64.600	< 0.001
≥ 8	4090	77.5%		
< 9	1990	67.9%	40.856	< 0.001
≥ 9	3804	77.6%		
< 10	2271	68.3%	52.554	< 0.001
≥ 10	3523	78.1%		
< 11	2517	69.3%	45.706	< 0.001
≥ 11	3277	78.0%		
< 12	2752	70.0%	39.887	< 0.001
≥ 12	3042	78.2%		
< 13	3005	70.0%	26.407	< 0.001
≥ 13	2789	78.7%		
< 14	3255	70.3%	17.277	< 0.001
≥ 14	2539	79.4%		
< 15	3459	71.0%	34.280	< 0.001
≥ 15	2335	79.1%		
< 16	3664	71.3%	31.059	< 0.001
≥ 16	2130	79.2%		
< 17	3778	71.4%	32.499	< 0.001
≥ 17	1916	79.9%		
< 18	4071	71.9%	24.916	< 0.001
≥ 18	1723	79.6%		
< 19	4237	72.3%	32.499	< 0.001
≥ 19	1557	79.2%		
< 20	4372	71.9%	24.916	< 0.001
≥ 20	1422	79.4%		
< 21	4531	72.7%	24.916	< 0.001
≥ 21	1263	79.4%		
< 22	4531	72.8%	13.907	< 0.001
≥ 22	1263	79.5%		

## DISCUSSION

Previously, several studies investigated the prognostic factors for node-negative gastric adenocarcinoma with R0 resection. In contrast to previous studies, we first used X-tile program to divided patients into high, middle, and low risk groups, and validated retrieved LNs count was an independent prognostic factor in node-negative gastric cancer. Then, in order to find minimum number of LNs that should be retrieved, we analyzed individual LN counts from 2 to 22 and found number of 14 met the criteria. After the number of 14, the 5-year CSS the survival rates were roughly equal. In addition, to the best of our knowledge, our study is the largest scaled one that assess lymph node count on prognosis in node-negative gastric cancer.

Compared with node-positive gastric cancer, node-negative tumors have less aggressive biological features and a more favorable prognosis [2, 6, 9]. However, patients with node-negative gastric cancer also experience recurrences [4, 10]. Identifying the risk factors associated with the recurrence of node-negative gastric cancer is important because these patients are assured to undergo radical resection and not have any microscopic regional metastases. LN retrieval is an independent risk factor for tumor recurrence and a poor prognostic factor in various cancer undergoing radical resection [11–14]. In the present study, we also found that the survival rates increased as the number of retrieved LN counts increased. There was 14.9% increase in 5-year CSS if ≥ 14 LN was retrieved compared with those with < 2 LN retrieved. Despite this correlation, the mechanism underlying the relationship between the number of LNs and survival has not been determined, although several hypotheses have been proposed.

The first hypothesis involves stage-migration. The isolated tumor cells and micrometastasis in negative LNs are considered as the key factors that could lead to the adverse effect on the overall survival of gastric cancer patients [15–17]. Harrison *et al*. demonstrated that patients with T3N0M0 gastric cancer who underwent extended lymphadenectomy had significantly more negative LNs than those who underwent limited lymphadenectomy, thereby indicating that extended lymphadenectomy can improve the overall survival of T3N0M0 patients. This result is potentially associated with the elimination of micrommetastasis in LNs [18]. Increased LNs retrieval will reduce the chance of understaging, and then improve survival.

The second hypothesis regards the function of LNs. It is considered that the immunity exerted by tumor-draining LNs is dual function for cancer cells, namely antitumor immunity and tolerance for cancer. Resection of regional LNs might reset the immunological balance, resulting in an improvement of patients' prognosis. A higher number of LNs dissected may simply reflect a host lymphocytic reaction to the tumor, which is associated with LN count [19], and lymphocytic reaction to tumor cells has been associated with longer survival in colorectal cancer [20, 21].

The second hypothesis revolves around the notion that the surgeon is a technician [22]. In theory, an increasing number of examined LNs indicates a high quality of surgical care. Improved surgical techniques may be the result of improved intraoperative staging [23], and therefore reduce the chances of iatrogenic spread of cancer cells [22]. As such, there is less likelihood of leaving tumor cells behind, thus positively affecting survival. Serosal exposure was an important prognostic factor for node-negative advanced gastric cancer after radical resection [24–26]. The chance of iatrogenic spread of cancer cells may be high in those patients. High quality of surgery will significantly reduce such adverse effect.

We acknowledge that our study suffers from several shortcomings. First, we only assessed CSS as end point in this study, for there were no information about local recurrence and distance metastasis in SEER database. Second, the SEER database does not include information regarding the administration of chemotherapy and the quality of surgical care or pathological technique, and all of these factors may impact survival outcomes, and we cannot adjust these factors in survival analysis. Third, distant LN metastases always classified as distant metastases and therefore the surgery were regarded as palliative resection, and should be excluded from this study. But for SEER data lacks such information, we cannot adjust for this.

In conclusion, retrieved LNs count was associated with long-time survival outcomes in node-negative gastric cancer. Patients would have a decrease in the risk of death for each additional LN examined up to a total of 14. In light of the association of LNs retrieval with postoperative treatment and prognosis, efforts to improve quality of care in this area could produce substantial improvements in outcome.

## MATERIALS AND METHODS

Data was obtained from the current SEER database (Surveillance Research Program, National Cancer Institute SEER*Stat software [version 8.2.1], http://www.seer.cancer.gov/seerstat) maintained by the National Cancer Institute, which consists of 18 population-based cancer registries. Inclusion criteria included the following: (1) patients were diagnosed from 2004 to 2012; (2) the site code was limited to stomach; (3) histology code was limited to adenocarcinoma (8140/3, 8144/3, 8255/3, 8211/3, 8260/3,8263/3), mucinous adenocarcinoma (8480/3), and signet ring cell carcinoma (8490/3); (4) with no LN metastasis(N0) and distant metastasis(M0); (5) underwent surgical resection; (6) at least with one LN retrieval; (7) age > 18 years old; (8) gastric cancer was the only one primary or first of more than one primary; (9) information on CSS and survival months available.

Standard patients' demographic, clinicopathologic data including histological type, tumor grade, T stage, number of LNs retrieval, and number of positive LNs were collected. Among these factors, sex, race, tumor grade, histologic type, T or N stage, primary site and tumor metastatic status were considered categorical variables. Continuous variables including age and retrieved LN count were categorized. We divided the patients into two age groups: < 60 years old and ≥ 60 years old. Patients were stratified by retrieved LN count into three groups according to cutoffs determined by X-tile program. All patients were restaged according to AJCC/UICC 7th edition.

### Statistical analysis

Statistics analysis was carried out using the statistical software package SPSS for Windows, version 21 (IBM Corp, Armonk, NY, USA). The LNs cutoff points were determined using the X-tile program (http://www.tissuearray.org/rimmlab/), which identified the cutoff with the minimum *P* values from log-rank χ^2^ statistics for the categorical LNs in terms of survival [8, 27]. A comparison of the categorical variables between LNs count subgroups was conducted using Pearson's χ^2^ test. Continuous variables were compared using the Mann-Whitney *U* test. To evaluate potential factors affecting survival time, taking survival time and censoring into account, Cox proportional hazards regression was used to report hazard ratio (HR) with 95% confidence intervals.

## References

[1] Siegel RL, Miller KD, Jemal A (2016). Cancer statistics, 2016. CA Cancer J Clin.

[2] Jian-Hui C, Shi-Rong C, Hui W, Si-le C, Jian-Bo X, Er-Tao Z, Chuang-Qi C, Yu-Long H (2016). Prognostic value of three different lymph node staging systems in the survival of patients with gastric cancer following D2 lymphadenectomy. Tumour Biol.

[3] Toth D, Plosz J, Torok M (2016). Clinical significance of lymphadenectomy in patients with gastric cancer. World J Gastrointest Oncol.

[4] Kooby DA, Suriawinata A, Klimstra DS, Brennan MF, Karpeh MS (2003). Biologic predictors of survival in node-negative gastric cancer. Ann Surg.

[5] Hyung WJ, Lee JH, Choi SH, Min JS, Noh SH (2002). Prognostic impact of lymphatic and/or blood vessel invasion in patients with node-negative advanced gastric cancer. Ann Surg Oncol.

[6] Xu D, Huang Y, Geng Q, Guan Y, Li Y, Wang W, Yuan S, Sun X, Chen Y, Li W, Zhou Z, Zhan Y (2012). Effect of lymph node number on survival of patients with lymph node-negative gastric cancer according to the 7th edition UICC TNM system. PLoS One.

[7] Deng J, Zhang R, Zhang L, Liu Y, Hao X, Liang H (2013). Negative node count improvement prognostic prediction of the seventh edition of the TNM classification for gastric cancer. PLoS One.

[8] Camp RL, Dolled-Filhart M, Rimm DL (2004). X-tile: a new bio-informatics tool for biomarker assessment and outcome-based cut-point optimization. Clin Cancer Res.

[9] Hsieh FJ, Wang YC, Hsu JT, Liu KH, Yeh CN (2012). Clinicopathological features and prognostic factors of gastric cancer patients aged 40 years or younger. J Surg Oncol.

[10] Huang KH, Chen JH, Wu CW, Lo SS, Hsieh MC, Li AF, Lui WY (2009). Factors affecting recurrence in node-negative advanced gastric cancer. J Gastroenterol Hepatol.

[11] Li Q, Liang L, Gan L, Cai G, Li X, Cai S (2015). Effect of Lymph Node Count on Pathological Stage III Rectal Cancer with Preoperative Radiotherapy. Sci Rep.

[12] Kim SH, Chong JU, Lim JH, Choi GH, Kang CM, Choi JS, Lee WJ, Kim KS (2016). Optimal assessment of lymph node status in gallbladder cancer. Eur J Surg Oncol.

[13] Zhou R, Wu Z, Zhang J, Wang H, Su Y, Huang N, Shi M, Bin J, Liao Y, Liao W (2016). Clinical significance of accurate identification of lymph node status in distant metastatic gastric cancer. Oncotarget.

[14] Wu SG, Wang Y, Zhou J, Sun JY, Li FY, Lin HX, He ZY (2015). Number of negative lymph nodes should be considered for incorporation into staging for breast cancer. Am J Cancer Res.

[15] Yonemura Y, Endo Y, Hayashi I, Kawamura T, Yun HY, Bandou E (2007). Proliferative activity of micrometastases in the lymph nodes of patients with gastric cancer. Br J Surg.

[16] Yanagita S, Natsugoe S, Uenosono Y, Kozono T, Ehi K, Arigami T, Arima H, Ishigami S, Aikou T (2008). Sentinel node micrometastases have high proliferative potential in gastric cancer. J Surg Res.

[17] Kim JH, Park JM, Jung CW, Park SS, Kim SJ, Mok YJ, Kim CS, Chae YS, Bae JW (2008). The significances of lymph node micrometastasis and its correlation with E-cadherin expression in pT1-T3N0 gastric adenocarcinoma. J Surg Oncol.

[18] Harrison LE, Karpeh MS, Brennan MF (1998). Extended lymphadenectomy is associated with a survival benefit for node-negative gastric cancer. J Gastrointest Surg.

[19] George S, Primrose J, Talbot R, Smith J, Mullee M, Bailey D, du Boulay C, Jordan H (2006). Will Rogers revisited: prospective observational study of survival of 3592 patients with colorectal cancer according to number of nodes examined by pathologists. Br J Cancer.

[20] Pages F, Galon J, Fridman WH (2008). The essential role of the *in situ* immune reaction in human colorectal cancer. J Leukoc Biol.

[21] Morris M, Platell C, Iacopetta B (2008). Tumor-infiltrating lymphocytes and perforation in colon cancer predict positive response to 5-fluorouracil chemotherapy. Clin Cancer Res.

[22] Li Q, Zhuo C, Cai G, Li D, Liang L, Cai S (2014). Increased number of negative lymph nodes is associated with improved cancer specific survival in pathological IIIB and IIIC rectal cancer treated with preoperative radiotherapy. Oncotarget.

[23] Le Voyer TE, Sigurdson ER, Hanlon AL, Mayer RJ, Macdonald JS, Catalano PJ, Haller DG (2003). Colon cancer survival is associated with increasing number of lymph nodes analyzed: a secondary survey of intergroup trial INT-0089. J Clin Oncol.

[24] Bruno L, Nesi G, Montinaro F, Carassale G, Boddi V, Bechi P, Cortesini C (2000). Clinicopathologic characteristics and outcome indicators in node-negative gastric cancer. J Surg Oncol.

[25] Adachi Y, Mori M, Maehara Y, Kitano S, Sugimachi K (1997). Prognostic factors of node-negative gastric carcinoma: univariate and multivariate analyses. J Am Coll Surg.

[26] Maehara Y, Tomoda M, Tomisaki S, Ohmori M, Baba H, Akazawa K, Sugimachi K (1997). Surgical treatment and outcome for node-negative gastric cancer. Surgery.

[27] Li QG, Li DW, Zhuo CH, Cai GX, Cai SJ (2014). Metastatic lymph node ratio can further stratify prognosis in rectal cancer patients treated with preoperative radiotherapy: a population-based analysis. Tumour Biol.

